# Anti-staphylococcus Antibiotics Interfere With the Transcription of Leucocidin ED Gene in *Staphylococcus aureus* Strain Newman

**DOI:** 10.3389/fmicb.2020.00265

**Published:** 2020-03-05

**Authors:** Han Yang, Su Xu, Kaifeng Huang, Xiaogang Xu, Fupin Hu, Chunyan He, Wen Shu, Zhiyan Wang, Fang Gong, Chuanling Zhang, Qingzhong Liu

**Affiliations:** ^1^Department of Clinical Laboratory, Shanghai General Hospital, Shanghai Jiao Tong University School of Medicine, Shanghai, China; ^2^Institute of Antibiotics, Huashan Hospital, Fudan University, Shanghai, China; ^3^Department of Cardiology, Shanghai General Hospital, Shanghai Jiao Tong University School of Medicine, Shanghai, China; ^4^Department of Clinical Laboratory, The Third Hospital Affiliated to Nantong University, Wuxi, China; ^5^Department of Clinical Laboratory, Xiaoshan Hospital, Hangzhou, China

**Keywords:** *Staphylococcus aureus*, leucocidin ED, *RNAIII*, antibiotic exposure, transcription

## Abstract

Antibiotics have been described to modulate bacterial virulence gene expression. This study aimed to assess the changes caused by anti-*Staphylococcus* agents in the transcription of leucocidin ED (*lukED*) gene of *Staphylococcus aureus* strain Newman *in vitro* and *in vivo* and to determine whether the altered expression is *agr* dependent. The bacteria were exposed to subinhibitory concentrations [1/2, 1/4, or 1/8 minimal inhibitory concentration (MIC)] of 11 antibiotics, and the expression of *lukE* and *agr*-effector RNAIII was determined using qRT-PCR. *In vivo* experiments were performed to evaluate the impact exerted by six representative antibiotics on the transcription of both genes. Molecular analysis showed that *in vitro lukE* transcription was dramatically promoted in the Newman strain exposed to sub-MICs of vancomycin, trimethoprim–sulfamethoxazole, clindamycin, gentamicin, daptomycin, and ciprofloxacin and considerably reduced when stimulated by cefazolin, erythromycin, rifampicin, tigecycline, and linezolid. In the murine abscess model, tigecycline significantly decreased the transcription of *lukE* and the bacterial numbers, whereas vancomycin increased them; although cefazolin increased the *lukE* expression (contrary to the *in vitro* effect), it had a remarkable role in reducing bacterial load. The correspondence analysis shows that *RNAIII* expression varied under seven of 11 antibiotics *in vitro*, and six drugs *in vivo* were consistent with *lukE* transcripts. In conclusion, our data show that anti-*Staphylococcus* antibiotics exert modulatory effects on *lukE* expression *in vitro* and/or *in vivo*, and the changed expression caused by some drugs may be involved with *agr* activity, thus providing a guide to choose appropriate agents to avoid promoting bacterial virulence in *lukED*-positive *S. aureus* infections.

## Introduction

*Staphylococcus aureus* is a pathogen notorious for its ability to cause many infection-related illnesses ranging from cutaneous infections and food poisoning to toxic shock syndrome, septicemia, and necrotizing pneumonia (Tong et al., 2015). The success of *S. aureus* infection stems from a repertoire of virulence factors that enable the bacteria to escape from the host immune system (Otto, 2014). Among these factors, leucocidin ED (LukED), a bicomponent pore-forming toxin, plays an important role in *S. aureus* pathogenicity (Alonzo and Torres, 2014; Balasubramanian et al., 2016).

LukED targets the membrane of various cells such as neutrophils, T cells, myeloid cells, macrophages, dendritic cells, and erythrocytes and elicits β-barrel pores that span the lipid bilayer and lead to osmotic lysis of the host cell (Alonzo et al., 2012, 2013; Reyes-Robles et al., 2013; Spaan et al., 2015). Epidemiological data and animal infection models show that *lukED* can be commonly detected in clinical *S. aureus* strains (approximately 2/3 to 4/5 of isolates) and is closely associated with impetigo, antibiotic-associated diarrhea, and bloodstream infection, among others (Gravet et al., 1998; Arciola et al., 2007; Alonzo et al., 2012; Alonzo and Torres, 2014; He et al., 2018). The accessory gene regulator (Agr)-repressor of toxin (Rot) pathway is an important modulatory network of LukED production (Alonzo et al., 2012). The *agr* operon encodes the regulatory RNA RNAIII, which promotes the transcription of leucocidin genes by negatively controlling the yield of Rot (Benson et al., 2014; Killikelly et al., 2015; Tan et al., 2018).

During treatment, bacteria may be exposed to subinhibitory levels [sub-minimal inhibitory concentrations (sub-MICs)] of antibiotics owing to drug-resistant organisms or the pharmacokinetics of antimicrobial agents (such as short half-life, poor drug distribution and adherence, or interactions between antibiotics) (Cars, 1990; Hodille et al., 2017). Early investigations have shown that sub-MICs of antibiotics may initiate differential expression of virulence genes in *S. aureus*, which may affect the pathogenesis of infection and result in worse outcomes (Dumitrescu et al., 2007, 2008, 2011; Stevens et al., 2007; Pichereau et al., 2012; Diep et al., 2013; Otto et al., 2013; Yamaki et al., 2013; Rudkin et al., 2014; Turner and Sriskandan, 2015; Hodille et al., 2017; Liu et al., 2018). Therefore, the therapeutic efficacy of antibiotics might also rely on their capacity to prevent the production of virulence factors (Otto et al., 2013). The use of antibiotics that reduce the Panton–Valentine leucocidin (PVL) toxin production is recommended for the treatment of severe infections caused by *pvl*-positive *S. aureus* (HPA, 2008; Nathwani et al., 2008). Nevertheless, little is known about the influence of antibiotics on *lukED* expression.

In this study, we selected common anti-*Staphylococcus* drugs to evaluate their impact on the expression of *lukED* in the *S. aureus* strain Newman *in vitro* and *in vivo*. We also analyzed whether the production of RNAIII is associated with variations in the levels of *lukED* transcripts affected by antimicrobial compounds.

## Materials and Methods

### Bacterial Strain and Culture Conditions

*Staphylococcus aureus* strain Newman was cultured at 37°C in yeast extract-Casamino Acids-pyruvate (YCP) medium [3% (w/v) yeast extract (Oxoid), 2% (w/v) casamino acids (Amresco, Washington, DC, United States), 2% (w/v) sodium pyruvate (Sangon Biotech, Shanghai, China), 0.25% (w/v) Na_2_HPO_4_, and 0.042% (w/v) KH_2_PO_4_, pH 7.0)], which is able to promote the highest expression of LukED (Alonzo and Torres, 2014).

### Antibiotics

The antimicrobials utilized in this work were cefazolin, gentamicin, erythromycin, tigecycline, rifampicin, daptomycin (purchased from Dalian Meilun Biotech, Dalin, China), ciprofloxacin, clindamycin, vancomycin (from the National Institutes for Food and Drug Control, Beijing, China), linezolid (Selleck Chemicals, Houston, TX, United States), and trimethoprim–sulfamethoxazole (Sigma–Aldrich, St Louis, MO, United States).

### Determination of Minimal Inhibitory Concentration

Minimal inhibitory concentrations of antibiotics against the *S. aureus* strain Newman were determined in triplicate by the standard microdilution broth method according to Clinical and Laboratory Standards Institute (CLSI) recommendations (Wayne, 2017).

### Growth Kinetics

Overnight liquid cultures of strain Newman were diluted 1:100 into 25 ml of fresh YCP medium, followed by addition of 1/8 MIC, 1/4 MIC, or 1/2 MIC antibiotics. Cultures without antibiotic served as control. Cultures were grown at 37°C with shaking at 150 r/min. Cell growth was detected by measuring the optical density (OD) at 600 nm every hour using a UV-2102C ultraviolet spectrophotometer (Unico Instruments, Shanghai, China).

### *In vitro* Exposure to Antibacterial Agents

Bacterial culture aliquots for RNA extraction were collected after the early (3 h) and late (5 h) logarithmic growth phases, when transcription of *lukED* was rising and reached the highest level, respectively (Yang et al., 2019).

### Extraction of Bacterial RNA

Bacterial culture samples were centrifuged at 13,000 × *g* and 4°C for 10 min; resuspended in TE buffer (10 mM of Tris HCl and 1 mM of EDTA, pH 8.0) with lysostaphin (1 mg/ml, Sangon Biotech, Shanghai, China) and proteinase K (20 mg/ml, TaKaRa, Dalian, China); and incubated at 56°C for 1 h for cell wall lysis. Total RNA was extracted using the MiniBEST Universal RNA Extraction Kit (TaKaRa, Dalian, China) according to the manufacturer’s instructions.

### Subcutaneous Abscess in Mice

Female Balb/c nude (nu/nu) mice between 4 and 6 weeks old were prepared for the abscess model. Mice were anesthetized with isoflurane and then injected subcutaneously with 100 μl of phosphate-buffered saline (PBS) containing 3 × 10^8^ colony-forming units (CFU)/ml of fresh Newman strain to form the abscess (Turner and Sriskandan, 2015).

### *In vivo* Exposure to Antimicrobials

After 48 h of infection, mice were injected intraperitoneally with 150 μl of 10 mg/kg of clindamycin, 10 mg/kg of linezolid, 25 mg/kg of cefazolin, 30 mg/kg of vancomycin, 4 mg/kg of daptomycin, 1.6 mg/kg of tigecycline, or PBS as a control according to the human therapeutic doses recommended by the *Sanford Guide to Antimicrobial Therapy* (Gilbert, 2014).

### Enumeration and RNA Extraction of Bacteria From Abscess

Following a previously described method (Turner and Sriskandan, 2015), mice were sacrificed after 8 h of treatment, and the abscess tissue was cut. Samples were diluted in PBS and plated for bacteria counting. The remaining abscesses were placed into liquid nitrogen quickly, followed by grinding for extraction of RNA as described above.

### Relative Quantitative RT-PCR

Bacterial RNA was quantified using a NanoDrop spectrometer (Thermo Fisher Scientific, Waltham, MA, United States), followed by purification and reverse transcription (1 μg of RNA) using the PrimeScript^TM^ RT reagent Kit with gDNA Eraser (TaKaRa, Dalian, China). Gene transcript levels were determined by quantitative real-time amplification (qRT-PCR, SYBR Premix Ex TaqTM, TaKaRa, Dalian, China) in a 7500 Real Time PCR System (Applied Biosystems, CA, United States). Primers for qRT-PCR are listed in Table 1 (Balasubramanian et al., 2016; Gaupp et al., 2016). The mRNA levels of target genes were standardized against those of the housekeeping gene *16S rRNA*. The fold change was determined using the 2^–ΔΔ*CT*^ method (Livak and Schmittgen, 2001).

**TABLE 1:** Primers used for quantitative RT-PCR (qRT-PCR) in this study.

**Primer**	**Sequence (5′–3′)**	**References**
*lukE*-F	GAAATGGGGCGTTACTCAAA	Balasubramanian et al., 2016
*lukE*-R	GAATGGCCAAATCATTCGTT	Balasubramanian et al., 2016
*RNAIII*-F	AGGAGTGATTTCAATGGCACAAG	Gaupp et al., 2016
*RNAIII*-R	TGTGTCGATAATCCATTTTACTAAGTCA	Gaupp et al., 2016
*16S rRNA*-F	CGTGCTACAATGGACAATACAAA	Gaupp et al., 2016
*16S rRNA*-R	ATCTACGATTACTAGCGATTCCA	Gaupp et al., 2016

### Statistical Analysis

One-way analysis of variance (ANOVA) followed by *a posteriori* Dunnett’s test was used to analyze the results (SAS Institute, Cary, NC, United States). Results were considered statistically significant when *p* < 0.05.

## Results

### Minimum Inhibitory Concentrations of Antibiotics

The MIC values of 11 antibiotics, summarized in Table 2, showed that the *S. aureus* strain Newman was susceptible to all the drugs tested.

**TABLE 2:** Minimal inhibitory concentrations (MICs) of 11 antibiotics for *Staphylococcus aureus* Newman.

**Antibiotic**	**MIC (μg/ml)**
Cefazolin (CFZ)	0.25
Gentamicin (GEN)	0.5
Ciprofloxacin (CIP)	0.5
Erythromycin (ERY)	0.5
Tigecycline (TGC)	0.25
Clindamycin (CLI)	0.125
Vancomycin (VAN)	2
Linezolid (LZD)	2
Rifampicin (RIF)	0.03
Daptomycin (DAP)	0.5
Trimethoprim–sulfamethoxazole (SXT)	1

### Impacts of Sub-Minimum Inhibitory Concentrations of Antibiotics on *Staphylococcus aureus* Growth

**FIGURE 1:**
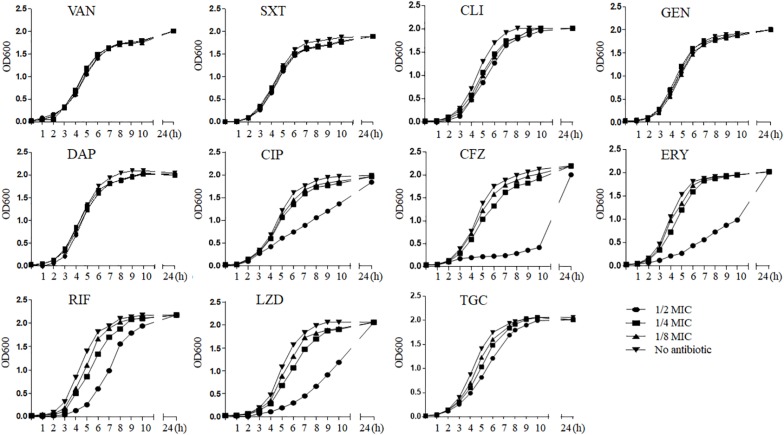
The influence of sub-MICs antibiotics on *Staphylococcus aureus* strain Newman kinetic growth. VAN, vancomycin; SXT, trimethoprim–sulfamethoxazole; CLI, clindamycin; GEN, gentamicin; DAP, daptomycin; CIP, ciprofloxacin; CFZ, cefazolin; ERY, erythromycin; RIF, rifampicin; LZD, linezolid; TGC, tigecycline; MIC, minimum inhibitory concentration.

We generalized the impact of 11 antibiotics at sub-MICs on strain Newman growth (Figure 1). As can be seen, graded concentrations of vancomycin, trimethoprim–sulfamethoxazole, clindamycin, gentamicin, daptomycin, and tigecycline triggered no significant growth defects over the entire growth curves compared with those of the control without drugs; in contrast, ciprofloxacin, cefazolin, erythromycin, rifampicin, and linezolid caused growth inhibition (*p* < 0.05) at 1/2 MIC. Because of this inhibition, we excluded these five antibiotics at 1/2 MIC from subsequent experiments of *in vitro* measurement of transcription to eliminate possible effects from antibiotic-induced growth impairment.

### Impact of Antibiotics on *lukE* Expression

**FIGURE 2:**
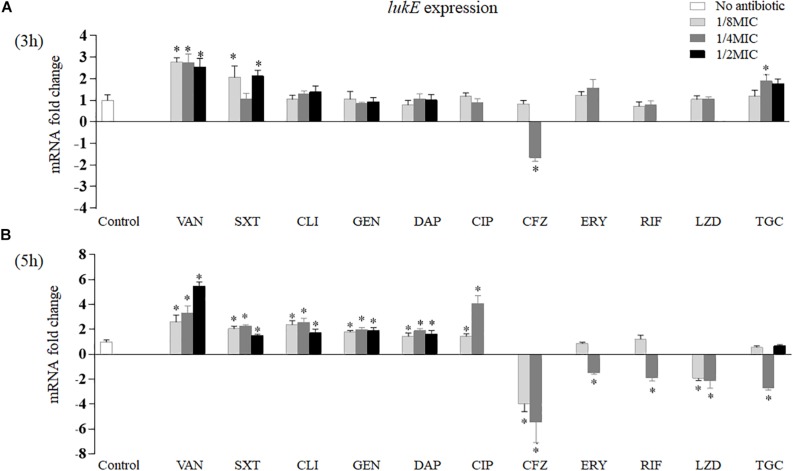
Impact of antibiotics at graded subinhibitory concentrations on the *in vitro* expression of *lukE* of *Staphylococcus aureus* Newman after 3 **(A)** and 5 h **(B)** of stimulation. Values are means ± SD (three technical replicates). ^∗^*p* < 0.05 compared with the no-antibiotic control. VAN, vancomycin; SXT, trimethoprim–sulfamethoxazole; CLI, clindamycin; GEN, gentamicin; DAP, daptomycin; CIP, ciprofloxacin; CFZ, cefazolin; ERY, erythromycin; RIF, rifampicin; LZD, linezolid; TGC, tigecycline.

As exhibited in Figure 2, after 3 h of *in vitro* incubation, only four of 11 antibiotics had effects on *lukE* expression (Figure 2A). Vancomycin at three sub-MICs detected significantly increased *lukE* transcription from 2.54- to 2.77-fold, respectively (*p* = 0.002 at 1/8 MIC, *p* = 0.004 at 1/4 MIC, and *p* = 0.006 at 1/2 MIC). Trimethoprim–sulfamethoxazole induced *lukE* mRNA production at 1/8 MIC (2.07-fold, *p* = 0.026) and 1/2 MIC (2.12-fold, *p* = 0.031). Tigecycline at 1/4 MIC enhanced *lukE* transcription level 1.89-fold (*p* = 0.019). In contrast, cefazolin also dramatically reduced the expression of *lukE* (1.65-fold, *p* = 0.037) at 1/4 MIC.

However, after 5 h of *in vitro* exposure, the 11 antibiotics examined all affected *lukE* mRNA transcription (Figure 2B). Treatment with vancomycin, trimethoprim–sulfamethoxazole, clindamycin, gentamicin, or daptomycin at all sub-MICs tested significantly increased *lukE* expression levels than did the no-drug control. Ciprofloxacin affected *lukE* mRNA levels particularly at 1/8 MIC and 1/4 MIC, ranging from 1.46- (*p* = 0.037) to 4.09-fold (*p* = 0.001), respectively. The transcript levels of *lukE* were considerably reduced in a concentration-dependent manner when exposed to 1/8 to 1/4 MICs of cefazolin (3.92-fold, *p* = 0.009 and 5.10-fold, *p* = 0.001, respectively). Strain Newman showed reduced *lukE* expression in the presence of 1/4 MIC of erythromycin (1.47-fold, *p* = 0.030) and rifampicin (1.88-fold, *p* = 0.010). Addition of 1/8 MIC (1.91-fold, *p* = 0.031) and 1/4 MIC (two-fold, *p* = 0.017) of linezolid and 1/4 MIC (2.71-fold, *p* = 0.003) of tigecycline led to reduced *lukE* transcript levels.

**FIGURE 3:**
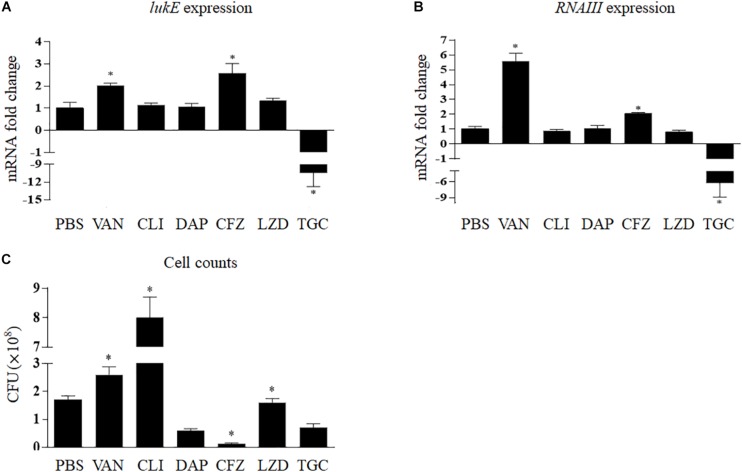
Impact of antibiotics on the expression of *lukE* **(A)** and *RNAIII* **(B)** and cell counts **(C)** of *Staphylococcus aureus* Newman within the abscess. Values are means ± SD (three technical replicates). *n* = 5 mice per group. ^∗^*p* < 0.05, compared with the group treated with PBS. TGC, tigecycline; CLI, clindamycin; DAP, daptomycin; LZD, linezolid; VAN, vancomycin; CFZ, cefazolin; PBS, phosphate-buffered saline.

Figure 3A shows that clindamycin, linezolid, and daptomycin had no relevant effects on *lukE* mRNA transcription *in vivo*; however, the expression of *lukE* was strikingly inhibited by tigecycline (10.10-fold, *p* < 0.001) and increased by vancomycin (2.03-fold, *p* = 0.009) and cefazolin (2.57-fold, *p* = 0.006). In addition, bacterial count results show that the total abscess bacterial load was significantly reduced by tigecycline, daptomycin, and cefazolin but considerably increased by clindamycin and vancomycin (Figure 3C).

### Impact of Antibiotics on *RNAIII* Expression

**FIGURE 4:**
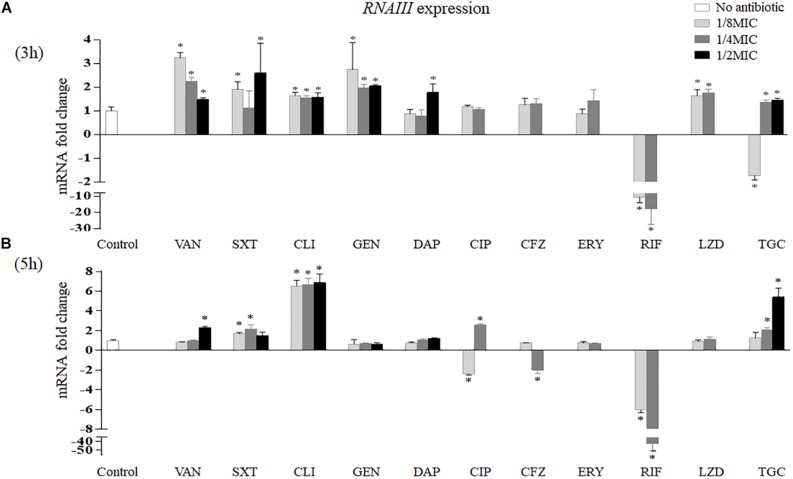
Impact of antibiotics at sub-MICs on the *in vitro* expression of *RNAIII* of *Staphylococcus aureus* Newman after 3 **(A)** and 5 h **(B)** of treatment. Values are means ± SD (three technical replicates). ^∗^*p* < 0.05 compared with the no-antibiotic control. VAN, vancomycin; SXT, trimethoprim–sulfamethoxazole; CLI, clindamycin; GEN, gentamicin; DAP, daptomycin; CIP, ciprofloxacin; CFZ, cefazolin; ERY, erythromycin; RIF, rifampicin; LZD, linezolid; TGC, tigecycline; MIC, minimum inhibitory concentration.

The effects of sub-MICs of antibiotics on *RNAIII* expression *in vitro* are shown in Figure 4. After 3 h of treatment, vancomycin induced *RNAIII* transcription at all sub-MICs tested (3.24-fold at 1/8 MIC, *p* < 0.001; 2.24-fold at 1/4 MIC, *p* = 0.001; and 1.47-fold at 1/2 MIC, *p* = 0.016). Trimethoprim–sulfamethoxazole increased *RNAIII* mRNA levels at 1/8 MIC (1.90-fold, *p* = 0.008) and 1/2 MIC (2.59-fold, *p* = 0.034). In addition, clindamycin and gentamicin all enhanced the expression of *RNAIII* at 1/8 MIC (1.65-fold, *p* = 0.008; 2.74-fold, *p* = 0.017), 1/4 MIC (1.55-fold, *p* = 0.010; 1.95-fold, *p* = 0.003), and 1/2 MIC (1.58-fold, *p* = 0.014; 2.04-fold, *p* = 0.002). Linezolid induced *RNAIII* expression by 1.63-fold at 1/8 MIC (*p* = 0.020) and 1.75-fold at 1/4 MIC (*p* = 0.006). *RNAIII* expression levels had a statistically significant increase at 1/2 MIC of daptomycin (1.77-fold, *p* = 0.018). Rifampicin reduced *RNAIII* expression by 9.90-fold at 1/8 MIC (*p* < 0.001) and 12.20-fold at 1/4 MIC (*p* = 0.004). Tigecycline reduced *RNAIII* expression by 1.72-fold at 1/8 MIC (*p* = 0.009) but enhanced its expression at 1/4 MIC (1.37-fold, *p* = 0.033) and 1/2 MIC (1.46-fold, *p* = 0.015) (Figure 4A).

After 5 h of treatment, *RNAIII* expression levels increased at 1/2 MIC of vancomycin (2.32-fold, *p* < 0.001), 1/8 MIC and 1/4 MIC of trimethoprim–sulfamethoxazole (1.68-fold, *p* = 0.002, and 2.14-fold, *p* = 0.004, respectively), and three sub-MICs of clindamycin (6.50-fold at 1/8 MIC, 6.67-fold at 1/4 MIC, and 6.86-fold at 1/2 MIC, *p* < 0.001). In contrast, *RNAIII* transcription decreased at 1/4 MIC of cefazolin (1.95-fold, *p* = 0.005) and sub-MICs of rifampicin (6.06-fold at 1/8 MIC and 41.67-fold at 1/4 MIC, *p* < 0.001). In addition, ciprofloxacin reduced the transcript levels of *RNAIII* at 1/8 MIC (2.40-fold, *p* < 0.001) but increased the expression of *RNAIII* at 1/4 MIC (2.56-fold, *p* < 0.001). Tigecycline increased *RNAIII* expression at 1/4 MIC (2.07-fold, *p* = 0.001) and 1/2 MIC (5.40-fold, *p* < 0.001) (Figure 4B).

*In vivo*, *RNAIII* transcript levels were remarkedly reduced by tigecycline by 5.37-fold (*p* = 0.004) and increased by vancomycin and cefazolin by 5.58-fold (*p* < 0.001) and 2.05-fold (*p* = 0.002), respectively (Figure 3B).

### Correspondence Analysis Between the Expression of *lukE* and *RNAIII*

Table 3 shows the correspondence between the transcription levels of *lukE* and *RNAIII in vitro* and *in vivo*. Our data demonstrate that the expressional variations of *RNAIII* had a consistent trend with those of *lukE* when exposed to clindamycin at 1/8 to 1/2 MICs for 5 h; tigecycline at 1/4 MIC for 3 h; vancomycin at 1/8 to 1/2 MICs for 3 h and 1/2 MIC for 5 h; trimethoprim–sulfamethoxazole at 1/8 MIC and 1/2 MIC for 3 h and at 1/8 MIC and 1/4 MIC for 5 h; and ciprofloxacin, cefazolin, and rifampicin at 1/4 MIC for 5 h. In the animal abscess model, the expression levels of *RNAIII* were strongly consistent with those of *lukE* after exposure to tigecycline, clindamycin, daptomycin, linezolid, vancomycin, and cefazolin.

**TABLE 3:** The correspondence of *RNAIII* and *lukE* expression in *Staphylococcus aureus* Newman after antibiotics exposure *in vivo* and *in vitro*.

**Antibiotic**	**Fold change in expression of *RNAIII* and *lukE in vivo***		**Subinhibitory concentration**	**Fold change in expression of**			
				***RNAIII* and *lukE in vitro***			
				**3 h of treatment**		**5 h of treatment**	
	***RNAIII***	***lukE***		***RNAIII***	***lukE***	***RNAIII***	***lukE***
Vancomycin	5.58	2.03	1/8 MIC	3.24	2.77	NC	2.61
			1/4 MIC	2.24	2.73	NC	3.31
			1/2 MIC	1.47	2.54	2.32	5.48
Trimethoprim–sulfamethoxazole	/	/	1/8 MIC	1.90	2.07	1.68	2.05
			1/4 MIC	NC	NC	2.14	2.29
			1/2 MIC	2.59	2.12	NC	1.52
Clindamycin	NC	NC	1/8 MIC	1.65	NC	6.50	2.40
			1/4 MIC	1.55	NC	6.67	2.58
			1/2 MIC	1.58	NC	6.86	1.73
Gentamicin	/	/	1/8 MIC	2.74	NC	NC	1.80
			1/4 MIC	1.95	NC	NC	1.98
			1/2 MIC	2.04	NC	NC	1.91
Daptomycin	NC	NC	1/8 MIC	NC	NC	NC	1.46
			1/4 MIC	NC	NC	NC	1.88
			1/2 MIC	1.77	NC	NC	1.64
Ciprofloxacin	/	/	1/8 MIC	NC	NC	−2.40	1.46
			1/4 MIC	NC	NC	2.56	4.09
			1/2 MIC	/	/	/	/
Cefazolin	2.05	2.57	1/8 MIC	NC	NC	NC	−3.92
			1/4 MIC	NC	−1.65	−1.95	−5.10
			1/2 MIC	/	/	/	/
Erythromycin	/	/	1/8 MIC	NC	NC	NC	NC
			1/4 MIC	NC	NC	NC	−1.47
			1/2 MIC	/	/	/	/
Rifampicin	/	/	1/8 MIC	−9.90	NC	−6.06	NC
			1/4 MIC	−12.20	NC	−41.67	−1.88
			1/2 MIC	/	/	/	/
Linezolid	NC	NC	1/8 MIC	1.63	NC	NC	−1.91
			1/4 MIC	1.75	NC	NC	−2.00
			1/2 MIC	/	/	/	/
Tigecycline	−5.37	−10.10	1/8 MIC	−1.72	NC	NC	NC
			1/4 MIC	1.37	1.89	2.07	−2.71
			1/2 MIC	1.46	NC	5.40	NC

*Data indicate increased or reduced fold variation (*p* < 0.05) in gene transcription compared with control without antibiotic. NC, no change; /, not detected; MIC, minimum inhibitory concentration.*

## Discussion

The *S. aureus* LukED toxin is able to trigger the damage of host cells and plays a vital role in controlling infection progress (Alonzo et al., 2013; Reyes-Robles et al., 2013; Spaan et al., 2015). Therefore, this toxin may be established as a novel potential target of antitoxin therapy for *S. aureus* diseases (Nocadello et al., 2016). Antimicrobial treatment for most infections can promote rapid bacterial damage. However, sometimes, elimination of the pathogen does not occur quickly enough to prevent the harmful impact of virulence factors (Hodille et al., 2017). Thus, antibiotics-mediated reduction of virulence factor production was suggested for the treatment of toxin-mediated diseases (Nathwani et al., 2008). Here, we explored the effects of anti-*Staphylococcus* antibiotics commonly used in the clinic on *lukED* expression *in vitro* and *in vivo* using the *S. aureus* strain Newman, a good producer of LukED.

Clindamycin, linezolid, erythromycin, gentamicin, and tigecycline, protein synthesis inhibitor compounds, block mRNA translation at the level of the ribosome to suppress the production of staphylococcal exotoxin protein (Hodille et al., 2017). Therefore, these drugs can exhibit broad anti-virulence traits. In this study, we discovered that these drugs at sub-MICs also modulated the mRNA levels (increase or decrease) of *lukE* (Figure 2). Possible interpretations for this observation are that protein synthesis inhibitors specifically disturb the expression of regulator(s) or two-component signal transduction system(s) that regulate transcription or translation of virulence determinants or that the activities of proteases and RNases affect the formation of the in-process product of the translational complex (Otto et al., 2013; Hodille et al., 2017). Previous reports showed that vancomycin has a minor effect on *pvl*, *hla*, and protein A (*spa*) mRNA levels (Dumitrescu et al., 2007, 2008; Otto et al., 2013; Hodille et al., 2017). Nevertheless, our findings exhibited a significant impact of vancomycin on *lukE* expression *in vitro*. This suggests that a cell wall-disrupting agent has the ability to induce some virulence gene expression at subinhibitory levels. It is believed that SOS response, leading to upregulation of an ensemble of DNA repair and recombination genes, can be activated by subinhibitory concentrations of trimethoprim and fluoroquinolones (Bisognano et al., 2004; Goerke et al., 2006; Hodille et al., 2017). In this investigation, the reason for the patently increased *lukE* expression regulated by trimethoprim–sulfamethoxazole and ciprofloxacin may be related to the SOS response. A previous study reported that transcription of *lukE* is remarkedly stimulated by low concentrations of penicillin and cefalotin (Subrt et al., 2011). However, sub-MICs of cefazolin strongly inhibited this gene transcription in this work. Cefalotin and cefazolin both belong to the first-generation cephalosporins binding to penicillin-binding protein 1 (PBP-1). The PBP-1-specific blockage by β-lactams can also initiate the SOS response (Hodille et al., 2017). However, this SOS-based mechanism of gene activation does not seem suitable to explain our observations. Here, we demonstrate an increased effect and a reduced effect on *lukE* transcription when *S. aureus* was exposed to sub-MICs of daptomycin and rifampicin, respectively. A published study showed that daptomycin also induces *pvl* mRNA, but the effect on *hla* mRNA level is varied and strain dependent (Otto et al., 2013). Rifampicin inhibits bacteria by suppressing the synthesis of mRNA; therefore, it is not surprising that this drug had an anti-LukED effect at sub-MIC.

It is well known that *in vitro* treatment with antimicrobials does not sufficiently correlate to clinical exposure to drugs during disease. In contrast to the *in vitro* data, we measured a pronouncedly increased level of *lukE* transcript in mice exposed to cefazolin but a significant reduction in the *S. aureus* burden (Figures 2, 3). Exposure to the protein synthesis inhibitors clindamycin and linezolid or the lipopeptide antibiotic daptomycin *in vivo* (no effect) was also in contrast to the effects of antibiotics on the transcription of *lukE in vitro* (Figures 2, 3). However, tigecycline, also a protein synthesis inhibitor, not only inhibited *lukE* expression *in vitro* and *in vivo* but also reduced the bacterial load (Figures 2, 3). The same observation for tigecycline was also reported in a rat burn model (Nosanov et al., 2017). The superior ability of tigecycline *in vivo* may be correlated with tigecycline-induced differential modification of matrix metalloproteinase-9, which can recruit leukocytes to the site of infection for the elimination of the bacteria (Simonetti et al., 2012).

Previous data from animal model showed that vancomycin was inferior to linezolid (Yanagihara et al., 2009; Martinez-Olondris et al., 2012). In the present investigation, we found that vancomycin had a poorer ability in reducing bacterial level and a stronger role in elevating *lukE* expression *in vivo* than had linezolid (Figures 2, 3). The increase or decrease in bacterial counts when using antibiotics may have a significant effect on the production of total virulence factors, which may affect the progress of disease. Therefore, simple *in vitro* experiments cannot accurately represent the final results, and thus more *in vivo* experiments are needed to evaluate the effect of antibiotics.

The expression of *S. aureus* virulence genes is controlled by complicated mechanisms (Pichereau et al., 2012). Many global modulators fine-tune virulence factor expression in response to outside signals such as host defenses and antibacterial agents. A regulator of this kind is the *agr* quorum-sensing system (Alonzo et al., 2012; Hodille et al., 2017). So far, there have been plenty of studies on the response of *agr* operon to antibiotics (Joo et al., 2010; Otto et al., 2013; Cazares-Dominguez et al., 2015; Jin et al., 2018). In those studies, the tested antibiotics, such as cephalosporins, penicillin, ciprofloxacin, tetracycline, clindamycin, and tigecycline, induced *RNAIII* transcription, but aminoglycosides and mupirocin had an inhibitory role. Here, we also detected the levels of *RNAIII* transcript. Table 3 shows that *agr* activity (*RNAIII* expression) was modified by 10 of the antibiotics tested (except erythromycin) in a concentration- and/or time-dependent manner *in vitro*, and *lukE* transcript levels varied under seven of them (except gentamicin, daptomycin, and linezolid) with a consistent trend. This suggests that most antibiotics tested at sub-MICs may modify *lukE* expression by affecting *agr* activity. Moreover, the *in vivo* experiments of six representative antibiotics also suggested the same conclusion (Figure 3B). Nevertheless, the mechanism by which antibiotics affect *agr* activity is unclear, and further investigation is needed. It is worth mentioning here that in our study, gentamicin, ciprofloxacin, cefazolin, and tigecycline did not show a completely consistent effect on *RNA III* expression, compared with those drugs mentioned by the above references. We speculate that the reason may be associated with the difference of antibiotic concentration, testing time point, and experimental strain.

The variations in *lukE* transcript levels may not necessarily translate to a difference in toxin production. Regrettably, we were not able to measure LukED toxin in the present study owing to the lack of a corresponding antibody. In addition, whether the *in vitro* and *in vivo* impacts on the Newman strain are applicable to other strains remains to be determined. Despite these shortcomings, our findings may still provide a clue to select suitable antibiotics for the treatment of *lukED*-positive *S. aureus* infections.

## Data Availability Statement

All datasets generated for this study are included in the article/supplementary material.

## Ethics Statement

The animal study was reviewed and approved by the Shanghai General Hospital ethical committee on animal experiments: 2018DW003.

## Author Contributions

HY, SX, and KH carried out the experiments and wrote the manuscript. XX and FH provided the laboratory for making experiments. CH, WS, and ZW analyzed the data and interpreted the results. FG and CZ revised the manuscript critically. QL designed the experiments and corrected the manuscript. All authors read and approved the manuscript.

## Conflict of Interest

The authors declare that the research was conducted in the absence of any commercial or financial relationships that could be construed as a potential conflict of interest.
